# Supplementary material for: Latest Developments in Minimally Invasive Spinal Treatment in Slovakia and Its Comparison with an Open Approach for the Treatment of Lumbar Degenerative Diseases
Source: J Clin Med. 2023 Jul 18;12(14):4755. doi: 10.3390/jcm12144755 (PMC10381332; doi:10.3390/jcm12144755)
Supplement: Supplementary file 1 [file jcm-12-04755-s001.zip › Supplementary material S2.pdf]

Supplementary material S2: Tests of Homogenity and Heterogeneity Measures

Supplementary material 2a: Tests of Homogenity and Heterogeneity Measures (Blood Loss)

Test of Homogeneity

	Chi-square (Q statistic)	df	Sig.
Overall	714,992	16	,000

Heterogeneity Measures

Overall	Tau-squared	42463,253
	H-squared	43,012
	I-squared (%)	97,7

Supplementary material 2b: Tests of Homogenity and Heterogeneity Measures (LOS)

Test of Homogeneity

	Chi-square (Q statistic)	df	Sig.
Overall	4420,336	13	,000

Heterogeneity Measures

Overall	Tau-squared	25,712
	H-squared	588,915
	I-squared (%)	99,8

Supplementary material 2c: Tests of Homogenity and Heterogeneity Measures (Operative time)

Test of Homogeneity

	Chi-square (Q statistic)	df	Sig.
Overall	637,156	16	,000

Heterogeneity Measures

Overall	Tau-squared	1269,375
	H-squared	42,198
	I-squared (%)	97,6

Supplementary material 2d: Tests of Homogenity and Heterogeneity Measures (ODI Change)

Test of Homogeneity

	Chi-square (Q statistic)	df	Sig.
Overall	116,364	13	,000

Heterogeneity Measures

Overall	Tau-squared	44,806
	H-squared	10,057
	I-squared (%)	90,1
