# Supplementary material for: Latest Developments in Minimally Invasive Spinal Treatment in Slovakia and Its Comparison with an Open Approach for the Treatment of Lumbar Degenerative Diseases
Source: J Clin Med. 2023 Jul 18;12(14):4755. doi: 10.3390/jcm12144755 (PMC10381332; doi:10.3390/jcm12144755)
Supplement: Supplementary file 1 [file jcm-12-04755-s001.zip › Supplemetary material S1.pdf]

# Supplemetary material S1: ODI Index Questinnaire

**Pateint name:**

We ask you to fill in the this questionnaire. The questionnarie is ment to monitor your health status before and after the operation. We will use your answers for the purpose of scientific study, which we will be publish in a scientific journal. Data will be anonymous and processed in accordance with GDPR.

Mark the answer that describes your current situation.

Thank you for filling out the questionnaire.

The author's collective.

## *Oswestry Disability Index*

### Section 1 – Pain Intensity

- ❑ I have no pain at the moment.
- ❑ The pain is very mild at the moment.
- ❑ The pain is moderate at the moment.
- ❑ The pain is fairly severe at the moment.
- ❑ The pain is very severe at the moment.
- ❑ The pain is the worst imaginable at the moment.

### Section 2 – Personal Care (washing, dressing, etc.)

- ❑ I can look after myself normally but it is very painful.
- ❑ I can look after myself normally but it is very painful.
- ❑ It is painful to look after myself and I am slow and careful.
- ❑ I need some help but manage most of my personal care.
- ❑ I need help every day in most aspects of my personal care.
- ❑ I need help every day in most aspects of self-care.
- ❑ I do not get dressed, wash with difficulty, and stay in bed.

### Section 3 - Lifting

- ❑ I can lift heavy weights without extra pain.
- ❑ I can lift heavy weights but it gives extra pain.
- ❑ Pain prevents me from lifting heavy weights off the floor, but I can manage if they are conveniently positioned (i.e. on a table).
- ❑ Pain prevents me from lifting heavy weights, but I can manage light to medium weights if they are conveniently positioned.
- ❑ I can lift only very light weights.
- ❑ I cannot lift or carry anything at all.

### Section 4 – Walking

- ❑ Pain does not prevent me walking any distance.
- ❑ Pain prevents me walking more than 1mile.
- ❑ Pain prevents me walking more than ¼ of a mile.
- ❑ Pain prevents me walking more than 100 yards.
- ❑ I can only walk using a stick or crutches.
- ❑ I am in bed most of the time and have to crawl to the toilet.

**Section 5 – Sitting**

- ❑ I can sit in any chair as long as I like.
- ❑ I can sit in my favorite chair as long as I like.
- ❑ Pain prevents me from sitting for more than 1 hour.
- ❑ Pain prevents me from sitting for more than ½ hour.
- ❑ Pain prevents me from sitting for more than 10 minutes.
- ❑ Pain prevents me from sitting at all.

**Section 6 – Standing**

- ❑ I can stand as long as I want without extra pain.
- ❑ I can stand as long as I want but it gives me extra pain.
- ❑ Pain prevents me from standing more than 1 hour.
- ❑ Pain prevents me from standing for more than ½ an hour.
- ❑ Pain prevents me from standing for more than 10 minutes.
- ❑ Pain prevents me from standing at all.

**Section 7 – Sleeping**

- ❑ My sleep is never disturbed by pain.
- ❑ My sleep is occasionally disturbed by pain.
- ❑ Because of pain, I have less than 6 hours sleep.
- ❑ Because of pain, I have less than 4 hours sleep.
- ❑ Because of pain, I have less than 2 hours sleep.
- ❑ Pain prevents me from sleeping at all.

**Section 8 – Sex life (if applicable)**

- ❑ My sex life is normal and causes no extra pain.
- ❑ My sex life is normal but causes some extra pain.
- ❑ My sex life is nearly normal but is very painful.
- ❑ My sex life is severely restricted by pain.
- ❑ My sex life is nearly absent because of pain.
- ❑ Pain prevents any sex life at all.

**Section 9 – Social Life**

- ❑ My social life is normal and cause me no extra pain.
- ❑ My social life is normal but increases the degree of pain.
- ❑ Pain has no significant effect on my social life apart from limitingmy more energetic interests, i.e. sports.
- ❑ Pain has restricted my social life and I do not go out as often.
- ❑ Pain has restricted social life to my home.
- ❑ I have no social life because of pain.

**Section 10 – Traveling**

- ❑ I can travel anywhere without pain.
- ❑ I can travel anywhere but it gives extra pain.
- ❑ Pain is bad but I manage journeys of over two hours.
- ❑ Pain restricts me to short necessary journeys under 30 minutes.
- ❑ Pain prevents me from traveling except to receive treatment.

**11. Did you undergo postoperative ambulant or institutional rehabilitation?**

YES NO

**12. Did you undergo postoperative spa treatment?**

YES NO

**13. Did the operation meet your expectations? Are you satisfied with it?** YES NO I don´t know

**SCORING TECHNIQUE FOR THE OSWESTRY LOW BACK DISABILITY QUESTIONNAIRE AND NECK DISABILITY INDEX**

1. Each of the 10 sections is scored separately (0 to 5 points each) and then added up (max. total = 50).

***Example:***

| Section 1. Pain Intensity | Point Value |
|---|---|
| A. ___ I have no pain at the moment | 0 |
| B. ___ The pain is very mild at the moment | 1 |
| C. ___ The pain is moderate at the moment | 2 |
| D. ___ The pain is fairly severe at the moment | 3 |
| E. ___ The pain is very severe at the moment | 4 |
| F. ___ The pain is the worst imaginable | 5 |

2. If all 10 sections are completed, simply double the patient's score.
3. If a section is omitted, divide the patient's total score by the number of sections completed times 5.

***Formula:***

$$\frac{\text{Patient's Score}}{\text{No. of sections completed x 5}} \text{ X } 100 = \_\_\_\_\_\_\_\_\_\_ \text{ \% DISABILITY}$$

***Example:***

If 9 of 10 sections are completed, divide the patient's score by 9 x 5 = 45.

Patient's Score 22

Number of sections completed: 9 (9 x 5 = 45)

22/45 x 100 = 48% disability

4. Interpretation of disability scores (from original article):

| **SCORE INTERPRETATION OF THE OSWESTRY LBP DISABILITY QUESTIONNAIRE** | |
|---|---|
| 0-20% Minimal disability | Can cope with most ADLs. Usually no treatment is needed, apart from advice on lifting, sitting, posture, physical fitness, and diet. In this group, some patients have particular difficulty with sitting and this may be important if their occupation is sedentary (typist, driver, etc.) |
| 20-40% Moderate disability | This group experiences more pain and problems with sitting, lifting, and standing. Travel and social life are more difficult and they may well be off work. Personal care, sexual activity, and sleeping are not grossly affected, and the back condition can usually be managed by conservative means. |
| 40-60% Severe disability | Pain remains the main problem in this group of patients, but travel, personal care, social life, sexual activity, and sleep are also affected. These patients require detailed investigation. |
| 60-80% Crippled | Back pain impinges on all aspects of these patients' lives both at home and at work. Positive intervention is required. |
| 80-100% | These patients are either bed-bound or exaggerating their symptoms. This can be evaluated by careful observation of the patient during the medical examination. |
| *Data compiled from Fairbanks et al, 1980.* | |
